# A Cost-Effective Standardized Quantitative Detection Method for Soil Microplastics in Different Substrates

**DOI:** 10.3390/toxics14010105

**Published:** 2026-01-22

**Authors:** Xinlei Ling, Yuting Gao, Rongxiang Li, Rongfang Chang, Yanpeng Li, Wen Xiao

**Affiliations:** 1 Institute of Eastern-Himalaya Biodiversity Research, Dali University, Dali 671003, China; lingxinlei0526@outlook.com (X.L.); m15752967410@163.com (Y.G.);; 2 Center for Interdisciplinary Sciences, Dali University, Dali 671003, China; 3 The Provincial Innovation Team of Biodiversity Conservation and Utility of the Three Parallel Rivers Region, Dali University, Dali 671003, China; 4 Collaborative Innovation Center for Biodiversity and Conservation in the Three Parallel Rivers Region of China, Dali 671003, China

**Keywords:** soil microplastics, standardized detection method, efficient detection, low-cost monitoring

## Abstract

Microplastics (MPs) are emerging pollutants with widespread global distribution, continuously accumulating in soils and posing risks of cross-media pollution. Current soil MP detection methods lack unified standards, suffering from high inter-laboratory variability and cost, which become key bottlenecks limiting data comparability and global microplastics pollution control. Here, we systematically reviewed soil MPs studies (2020–2024) and based on stepwise verification, we established a standardized, reproducible detection method: soil samples were dried at 80 °C for 12 h; density separation was performed in Erlenmeyer flasks with decantation, 10 s glass rod stirring, and 12 h settling, repeated five times; digestion was conducted using a 1:2 volume ratio of H_2_O_2_ to supernatant at 80 °C for 8 h; and MPs were quantified via stereo-microscopy combined with ImageJ. It should be noted that the use of NaCl limits the recovery of high-density polymers (e.g., PVC, PET), and the minimum detectable particle size is approximately 127 µm. The method was validated in sandy, loam, and clay soils, achieving an average recovery rate of 96.4%, with a processing time of 68 h and a cost of USD 9.77 per sample. In contrast to previous fragmented, non-standardized protocols, this workflow synergistically optimizes high recovery efficiency, cost-effectiveness, and broad applicability, offering a low-cost, efficient, and widely applicable approach for soil MPs monitoring, supporting data comparability across studies and contributing to global pollution assessment and the United Nations 2030 Sustainable Development Goals.

## 1. Introduction

Microplastics (MPs) refer to plastic particles with a particle size of less than 5 mm. As a novel environmental pollutant characterized by complex compositions and poor biodegradability, they have been widely distributed in various global environmental media, such as water bodies, soils, and the atmosphere [1,2]. Owing to their strong environmental persistence, bioaccumulation, and combined effects with organic pollutants, MPs not only pose a serious threat to ecosystem stability and biodiversity but also may exert adverse impacts on human health through food chain transmission [3,4,5,6]. At the second session of the United Nations Environment Assembly, microplastic pollution was listed as the second most important scientific challenge, second only to climate change [7].

MPs are characterized by their enormous quantity and diverse migration pathways and are widely distributed on a global scale. The large-scale production and use of plastic products have led to a continuous increase in plastic waste, with approximately 52.1 million tons of plastic waste generated globally each year [8]. It is predicted that by 2050, the global annual plastic production will reach 2.92 billion tons, and the amount of plastic waste will reach 245.2 million tons [9]. A large amount of plastic waste enters the environment due to improper management, and degrades and fragments under the action of physical, chemical, and biological processes, forming MPs particles that accumulate continuously in the environment [10]. Through sludge application to farmland alone, Europe and North America input 430,000 tons and 300,000 tons of MPs into agricultural soils each year, respectively [11]. Agricultural practices are important pathways driving the migration of MPs; for instance, residual plastic films can lead to the continuous release of MPs into the soil [12]. Meanwhile, soil organisms, such as earthworms and collembolans, can promote the horizontal diffusion and vertical downward movement of MPs in the soil through behaviors including burrowing, feeding, and excretion [13,14]. A large number of MPs remaining in the soil can enter the atmospheric environment through wind action [15], and then undergo local or long-distance transportation via wind and turbulence, enabling a global migration [16,17]. More MPs can enter aquatic environments such as rivers and lakes along with surface runoff and precipitation [18,19], and then return to the soil environment again through irrigation systems [20]. In general, MPs can cycle and migrate between environmental media such as water, soil, and atmosphere through human activities, biological processes, and abiotic processes, causing global cross-contamination [12,21]. Therefore, MPs have been detected in almost all monitored ecosystems, from polar ice sheets to deep-sea trenches and from mountain summits to agricultural topsoils, presenting an ‘omnipresent’ pollution pattern [22,23,24,25,26]. Facing this global environmental challenge, the development of scientific pollution prevention and control strategies urgently requires a systematic and accurate understanding of the distribution characteristics and migration patterns of MPs in the environment [27,28].

To reveal the distribution characteristics and migration patterns of MPs in the environment, global research teams have made considerable efforts and achieved certain progress [29,30,31]. Despite considerable progress, the current methodologies for MPs detection remain fragmented and inconsistent, which hinders global data comparability. First, at the methodological level, a unified standard for MPs detection has not yet been established [28,32]. Our statistical analysis of 73 studies published between 2020 and 2024 revealed wide variations and incomplete reporting in critical experimental parameters, including drying, density separation, and digestion steps (Table S1 in the Supplementary Materials). The results showed that there were significant differences in parameter reporting and operational procedures for key experimental steps among different studies. In the sample drying stage, the temperature setting ranged from room temperature to 90 °C, and the time span varied from 2 h to 35 days; most studies did not clearly report the drying time. During the density separation process, various types of containers were used; auxiliary dispersion methods included ultrasound, stirring, shaker agitation, and manual shaking, with treatment times ranging from 30 s to 2 h; standing time was between 6 and 48 h; the number of density separation cycles fluctuated from 1 to 6; and only 30% of the studies provided clear operational details for the supernatant extraction method. In the digestion stage, the temperature ranged from room temperature to 75 °C, and the digestion time varied from 5 min to 21 days; the missing rate of parameters for the ratio of supernatant to digestion solution reached 57%. In addition, the standardization of step sequence and detection workflow was also insufficient: most studies adopted the ‘density separation → digestion’ workflow, while some studies used other sequences such as ‘digestion → density separation’ or ‘density separation → digestion → density separation’. These inconsistencies in parameters and workflows directly affect the recovery efficiency of MPs as well as the comparability and reproducibility of detection results, restricting the integrated analysis of data and the accuracy of environmental risk assessment. Therefore, there is an urgent need to establish a set of standardized methods to improve the consistency and comparability of global data. Crucially, such standardization must account for matrix-specific challenges. Unlike the relatively homogeneous and less organic-rich marine or sediment environments for which many existing protocols were developed, soil presents a highly complex and heterogeneous matrix with significant organic matter, varied mineralogy, and strong particle aggregation. These factors substantially complicate MP isolation and recovery, necessitating tailored methods rather than direct adoption of aquatic-focused protocols [33,34]. Second, due to the large consumption of plastics and weak waste management, the problem of MPs pollution is particularly severe in developing countries, which urgently require a cost-effective detection method [35,36,37]. Currently, mainstream methods for MPs monitoring rely on expensive instrumental equipment, mainly including spectroscopic techniques and thermal analysis techniques [12]. Spectroscopic techniques, such as Fourier transform infrared spectroscopy (FTIR), Raman spectroscopy (Raman), and near-infrared spectroscopy (NIR), generally cost between USD 10,000 and USD 300,000. Thermal analysis techniques such as thermogravimetric analysis-mass spectrometry (TGA-MS), pyrolysis-gas chromatography-mass spectrometry (Pyr-GC-MS), and thermal extraction desorption–gas chromatography–mass spectrometry (TED-GC-MS) are more expensive, usually ranging from USD 200,000 to USD 600,000 [38,39,40]. Such high costs make it difficult for developing countries to afford the expenses of MPs detection. The absence of effective monitoring methods impedes the risk assessment, policy formulation, and pollution control of MPs, further exacerbating the pollution problem [32]. Such inconsistencies directly undermine recovery efficiency, reproducibility, and global data comparability. Further, to achieve the United Nations Sustainable Development Goals by 2030, there is an urgent need to develop a cost-effective MPs detection method suitable for developing countries [41].

In this study, we systematically reviewed existing methodologies, optimized key experimental parameters through stepwise verification, and established a standardized, low-cost workflow applicable across multiple soil types. This approach aims to improve global data comparability, support ecological risk assessments, and provide a technical foundation for sustainable soil pollution management.

## 2. Materials and Methods

### 2.1. Development of a Standardized Technical Route for Soil MPs Quantification

**Figure 1:**
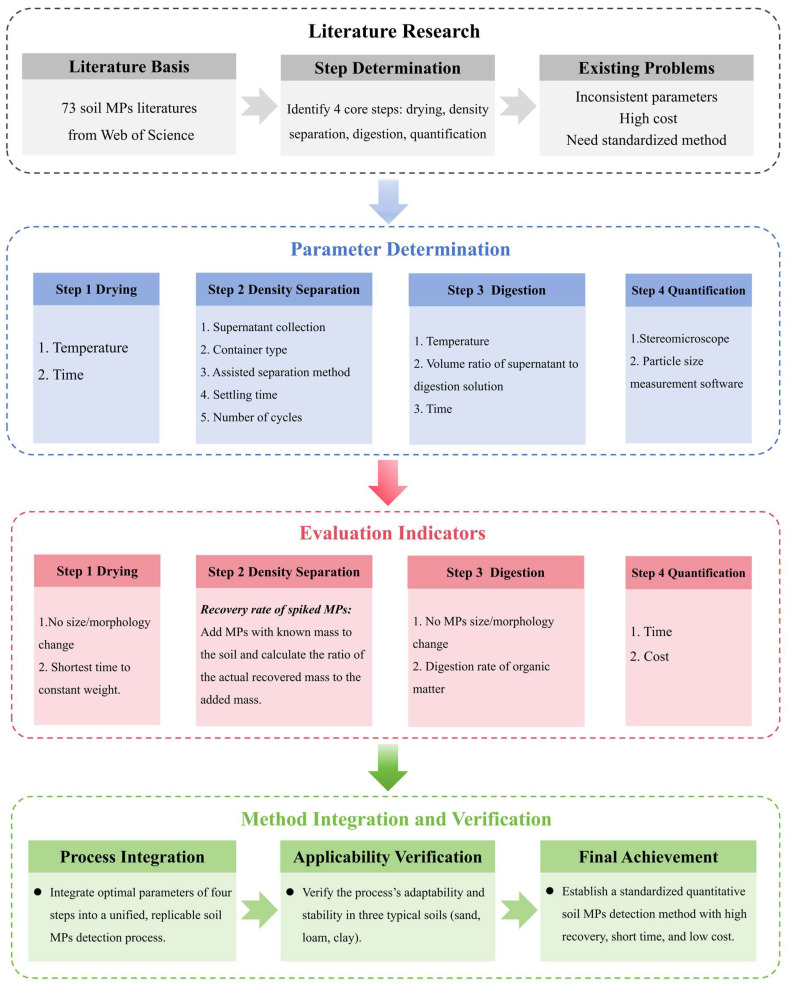
Technical roadmap for quantitative detection of MPs in soil.

In this study, a technical route for quantifying MPs in soil was devised (Figure 1).

### 2.2. Specific Experimental Procedures

#### 2.2.1. Soil Sample Collection and Preparation

Soil samples were collected from the foothills of Cangshan Mountain (25°40′ N, 100°09′ E) in Dali City, Dali Bai Autonomous Prefecture, Yunnan Province, China. The sampling depth was 0–20 cm for surface soil. To cover common soil particle composition types, three typical textural natural soils (sand, loam, and clay) were selected, as these three classes represent the most common natural soil types and can effectively reflect the separation behavior of MPs under different soil matrices. Approximately 10 kg of each soil type collected. After collection, the samples were passed through a 5 mm stainless steel sieve to remove gravel and plant residues, then sealed, stored in the dark, and kept in a refrigerator at 4 °C for later use. Before the experiment, the initial moisture content and mass of the soils were determined: the initial moisture content of sand was 9.74% with a mass of (52.13 ± 3.33) g; that of loam was 10.15% with a mass of (45.12 ± 1.28) g; and that of clay was 54.66% with a mass of (61.38 ± 3.42) g. Meanwhile, natural alpine meadow soil samples were collected synchronously from the study area. The flotation method (using a saturated NaCl solution (Sinopharm Chemical Reagent Co., Ltd., Shanghai, China) with a density of 1.2 g/cm^3^ and a temperature of 25 °C as the flotation agent) was employed to separate organic components. After drying at 80 °C, the samples were sieved through a 5 mm sieve to obtain background samples with high organic matter content, which were used for the subsequent determination of digestion parameters.

Polyethylene (PE), polypropylene (PP), and polystyrene (PS) microplastic particles, which are widely present in the environment, were selected as spiked MPs for the experiment. These particles were purchased from Kaixuan Plastic Technology Co., Ltd., Guangzhou, China, with an irregular morphology and a purity of ≥95%. They were divided into two particle size groups: the small particle size group (127.9–473.7 μm) and the large particle size group (1251–2012 μm). A unified naming format ‘polymer type-particle size group-MP’ was adopted, where ‘S’ represents small particle size and ‘L’ represents large particle size. For example, PE-S-MP denotes small-particle-size polyethylene MPs, and PS-L-MP denotes large-particle-size polystyrene MPs.

The preparation of spiked soil samples was carried out in two stages to ensure the systematic validation of the method. The first stage aimed to determine the parameters of core steps. Considering that loam has a moderate texture, which can reduce the interference of extreme soil textures on the initial experiment, the pre-treated loam was selected as the matrix. A 50 mL loam sample was measured, and 1.0 g of target MPs with a single type and single particle size was added to it. After thorough mixing, the mixture was transferred to an electric blast drying oven (Shanghai Yiheng Scientific Instrument Co., Ltd., Shanghai, China) and dried at 80 °C for 12 h to prepare spiked loam samples with a single variable, which were used for parameter screening of core steps such as density separation. The second stage focused on evaluating the adaptability of the method workflow. Two sets of spiking schemes were set up simultaneously, and the soil types were expanded to sand, loam, and clay. One set continued the single-spiking operation of the first stage, i.e., 1.0 g of MPs with a single type and single particle size was added to the soil. The other set added a mixed-spiking group: six types of MPs were selected, including PE, PP, and PS (each type contained both small and large particle size groups). For each type, 0.5 g was accurately weighed, and the total mass of the mixture was 3 g. All spiked soils were thoroughly mixed and then treated under the unified condition of drying at 80 °C for 12 h. Finally, spiked samples of the three soil types were obtained. All experiments in this study were conducted with 5 parallel samples.

#### 2.2.2. Optimization of Key Parameters and Method Validation

To optimize the standardized workflow, we systematically validated the critical steps, including drying, density separation, organic matter digestion, sequence of density separation and digestion, and quantitative analysis.

(i) Drying Parameters

In the preliminary experiment, MPs derived from common plastic products (e.g., supermarket bags, disposable gloves, food packaging) were subjected to heat treatment within the temperature range of 25–100 °C after cutting, grinding, and sieving. Observations via ultra-depth 3D microscopy revealed that no obvious thermal deformation was observed at temperatures up to 80 °C. However, when the temperature reached 90 °C, the samples began to exhibit edge curling and surface collapse; at temperatures of 100 °C and above, melting or severe deformation occurred (Supplementary Figure S1). Therefore, in the formal experiment of this study, six temperatures including the safe threshold of 80 °C and below were selected: 25 ± 1 °C (room temperature control), 50 °C, 60 °C, 70 °C, 80 °C, and 90 °C. During the experiment, the six types of MPs were evenly spread on clean glass plates and heated in an electric blast drying oven for 12 h; after cooling to room temperature, morphological images were uniformly captured using an ultra-depth 3D microscope at a magnification of 500×, and particle sizes were measured.

Based on the optimal drying temperature screened above, the minimum drying time for the three soil types (sand, loam, and clay) was further determined. For the experiment, 50 mL of each pre-treated soil sample (of the three types) was evenly spread in a glass petri dish with a diameter of 90 mm, and the petri dishes were then placed under the optimal temperature for continuous drying. Starting from 4 h after the initiation of drying, the mass of the soil was weighed using an electronic balance (OHAUS Instruments Co., Ltd., Changzhou, China) at 1-h intervals; when the difference between two consecutive weighing results was <0.01 g, the soil was considered constant weight, and the corresponding drying duration was recorded as the minimum drying time for that soil type.

(ii) Density Separation Parameters

Density separation is a core step in the extraction of MPs from soil. In this experiment, a saturated NaCl solution was used as the flotation liquid, and the spiked loam samples prepared in Section 2.2.1 were employed to investigate five key parameters: container type, supernatant collection method, auxiliary separation method, standing time, and number of density separation cycles. MPs recovery rate was used as the evaluation index, and the specific parameter determination protocols are as follows:

- Container type: Two types of containers (both with a volume of 250 mL) were tested, namely wide-mouth beakers and Erlenmeyer flasks.
- Supernatant collection method: Two supernatant collection methods were set up. The first was the pouring method: after standing, approximately 50 mL of supernatant was slowly poured at a 45° angle to avoid disturbing the sediment at the bottom. The second was the pipette extraction method: a 10 mL glass pipette was used to extract supernatant from 1 cm below the liquid surface, with a total extraction volume of 50 mL.
- Auxiliary separation method: In the preliminary experiment, two auxiliary separation methods (shaker oscillation and ultrasonic treatment) were tested at four durations (5 min, 10 min, 20 min, and 30 min), with MPs recovery rate as the evaluation index for comparison. The results showed that ultrasonic treatment achieved the optimal recovery rate at 10 min, while shaker oscillation reached the highest recovery rate at 20 min (Supplementary Figure S2). Based on this, five auxiliary separation methods were set up in the formal experiment, including four treatment groups and one non-treatment control group: the shaker oscillation group (optimal duration of 20 min, frequency of 200 rpm); the ultrasonic treatment group (optimal duration of 10 min, frequency of 40 kHz); the hand-shaking group (holding the Erlenmeyer flask and oscillating for 5 s); the glass rod stirring group (stirring with a glass rod for 10 s); and the control group (only standing, without any physical disturbance).
- Standing time: Natural sedimentation was adopted in this stage, with four standing time gradients set: 6 h, 12 h, 24 h, and 48 h.
- Number of density separation cycles: Three gradients of density separation cycles were set: 3 cycles, 4 cycles, and 5 cycles. The operation process for a single cycle was as follows: adding saturated NaCl solution → thorough mixing → standing for stratification → collecting supernatant. It should be noted that fresh NaCl solution was replaced in each cycle to avoid fluctuations in solution concentration affecting buoyancy stability.

In addition, the supernatant from all groups was digested with 30% H_2_O_2_ (Sinopharm Chemical Reagent Co., Ltd., Shanghai, China) under digestion conditions of 80 °C for 12 h. After digestion, the solution was filtered by suction through a 0.45 μm aqueous membrane. The membrane was dried and weighed to calculate the MPs recovery rate.

(iii) Digestion Parameters

To efficiently remove organic matter from soil and avoid interfering with MPs detection, this study determined the key parameters of the H_2_O_2_ digestion system (digestion temperature, volume ratio of supernatant to H_2_O_2_, and digestion time) in three stages. The specific experimental design is as follows:

The same MPs as used in the previous experiments were selected. The six types of MPs were separately dispersed on clean glass plates, and 10 mL of 30% H_2_O_2_ solution was added to each plate. The plates were then placed at the set temperatures for continuous digestion for 12 h. Four temperature gradients were set in the experiment: 60 °C, 70 °C, 80 °C, and 90 °C. After digestion, the MPs were transferred to clean glass petri dishes, and their surface morphology was observed and particle sizes were measured using an ultra-depth 3D microscopy system (Keyence Corporation, Osaka, Japan).

To determine the optimal volume ratio of supernatant to H_2_O_2_, 1.0 g of high-organic-matter sample (prepared via the flotation method in Section 2.2.1) was mixed with 150 mL of saturated NaCl solution and allowed to stand for 12 h to simulate the supernatant system after density separation. Subsequently, H_2_O_2_ was added at different volume ratios (150 mL supernatant:30% H_2_O_2_), with five ratio gradients set: 3:1, 2:1, 1:1, 1:2, and 1:3. Digestion was carried out at the previously determined optimal temperature for 12 h. After the reaction, the mixture was filtered by suction through a 0.45 μm aqueous membrane. The membrane was dried and weighed to calculate the digestion rate of organic matter.

Based on the optimal temperature and optimal volume ratio, the digestion time was further optimized. A total of 1.0 g of high-organic-matter soil sample was weighed and mixed with 150 mL of saturated NaCl solution. After standing for 12 h, the required volume of 30% H_2_O_2_ was calculated according to the screened optimal volume ratio and added to the mixture, followed by continuous digestion at the optimal temperature. Seven time gradients were set: 4, 6, 8, 10, 12, 24, and 48 h. Samples were taken at each time point and subjected to subsequent treatments as described above.

(iv) Sequence of Density Separation and Digestion

This study compared the effects of three different procedural sequences on the recovery rate of MPs. The experiment was conducted based on the spiked soil samples prepared in the previous stage, and the specific treatment methods are detailed in Table 1.

(v) Quantitative Parameters

To screen the optimal microscope–software combination for the quantitative analysis of MPs, this study systematically evaluated the applicability of 3 types of microscopes and 3 types of analytical software. For the microscope evaluation, the same types and particle size ranges of MPs as used in previous studies were adopted, including a stereomicroscope (maximum magnification: 50×), a biological microscope (maximum magnification: 1000×), and a 3D ultra-depth microscope (maximum magnification: 2000×). The stereomicroscope was used to locate targets under low magnification, and a smartphone was aligned with its eyepiece to capture clear, distortion-free images for manual counting and morphological observation. After sample preparation, the biological microscope first located targets under low magnification and then observed them under high magnification, enabling manual counting and measurement of parameters such as particle size and perimeter. The 3D ultra-depth microscope performed observation through gradual magnification and contrast adjustment, and also supported manual counting and morphological parameter measurement.

For the software evaluation, real environmental MPs samples were used, and analysis was conducted based on images acquired by the 3D ultra-depth microscope. After calibration via the ‘Measurement → Scale Bar’ function in VHX software, the ‘Automatic Area Measurement’ module was used to identify particles; impurities were manually removed, and particle size and area data were exported (automatic measurement mode). In ImageJ software (version 1.4.3.67, developed by Broken Symmetry Software), calibration was completed by drawing a scale bar and inputting the actual length (e.g., 100 μm); a grayscale threshold (0–80) was set to separate targets from the background, and no fewer than 10 particles were manually selected to measure their maximum axis diameter (threshold segmentation + manual measurement mode). In Nano Measurer 1.2 software, calibration was performed by manually drawing a scale bar; the maximum axis diameter of at least 10 particles was measured one by one, and an analysis report was generated (manual measurement mode). Consequently, the selected ‘stereomicroscope + ImageJ’ approach is a morphological identification method suitable for high-throughput screening and quantitative monitoring, and does not provide polymer-specific chemical confirmation.

#### 2.2.3. Validation and Evaluation of the Standardized Procedure

Based on the key parameters of drying, density separation, digestion, and quantification determined in Section 2.2.2, this study established a complete detection procedure for MPs in soil and validated the applicability of this procedure in three different soil types (sand, loam, and clay). Various soil samples were processed in accordance with this procedure, and the robustness and applicability of the detection method were evaluated by determining the recovery rate of MPs.

To clarify the position of the standardized detection procedure established in this study among existing methods, a literature review was conducted to comparatively analyze the MPs detection methods in soil reported in recent years. The literature search was performed in the Web of Science Core Collection database (2019–2024) using a unified search formula: TS = (“microplastics*” AND “soil” AND “method*”). After screening the search results by title, abstract, and full text, 11 studies that met the following criteria were finally included: (i) explicitly providing the recovery rate data of artificially spiked MPs; (ii) recording the total operation time from sample pretreatment to the completion of detection in detail; (iii) listing the information of key reagents, consumables, and equipment. The basic information of the included literature is summarized in Supplementary Table S2.

### 2.3. Quality Assurance and Quality Control (QA/QC)

QA/QC procedures included strict contamination prevention, process blanks and airborne exposure tests, and spiked recovery validation.

(i) Strict contamination prevention: All experiments were conducted under contamination-minimized conditions. Plastic labware was systematically avoided; only glass and stainless-steel materials were used. Researchers wearing cotton lab coats and nitrile gloves. All glassware and metal tools were rinsed 3–4 times with deionized water filtered through a 0.45 μm membrane; reagents (NaCl) and ultrapure water were also pre-filtered through a 0.45 μm membrane. Thereby reducing the risk of contamination from airborne MPs deposition, all containers were covered with clean glass lids, and experiments were conducted in a closed laboratory to minimize airborne deposition.
(ii) Process blanks and airborne exposure tests: For each experimental batch, five procedural blanks and five airborne blanks (open-filter exposure) were included. Contamination was negligible: procedural blanks contained an average of 2 MPs (mainly fibers, 100–500 μm), and airborne blanks contained <1 MP on average, confirming minimal background interference.
(iii) Spiked recovery validation, slightly >100% recoveries occasionally occurred, likely due to MPs binding trace solvents/moisture or co-precipitating with inorganics during extraction. Similar phenomena have been reported in previous MPs studies [42], supporting the robustness of the method.

Overall, the negligible blank values and consistent recovery rates demonstrate the accuracy, reproducibility, and applicability of the established method.

### 2.4. Data Analysis

#### 2.4.1. Calculation of Key Indicators

(i) Recovery Rate of MPs

Recovered mass of MPs: *M_recovered_* = (*M_final_* − *M_initial_*) − *M_blank_*

where *M_final_* is the final constant mass of the filter membrane, *M_initial_* is the initial mass, and *M_blank_* is the mean value of the blank control group (*n* = 5);

$$\mathrm{Recovery}\;\mathrm{rate}\;\mathrm{of}\;\mathrm{MPs}\;=\frac{M_{\mathrm{recovered}}}{M_{\mathrm{added}}}\;\times \;100\%$$

where *M_recovered_* refers to the net mass of MPs actually recovered through the experimental process, and *M_added_* refers to the total mass of MPs added at the beginning of the experiment, which is 1.0000 g.

(ii) Digestion Rate of Organic Matter

$$\mathrm{Digestion}\;\mathrm{rate}\;\mathrm{of}\;\mathrm{organic}\;\mathrm{matter}=\frac{M_{\mathrm{pre}}-M_{\mathrm{post}}}{M_{\mathrm{pre}}-M_{\mathrm{membrane}\;\mathrm{blank}}}\;\times \;100\%$$

where *M_pre_* and *M_post_* are the masses of the filter membrane before and after digestion, respectively; *M_membrane_* _*blank*_ is the mass of the blank filter membrane (without any residues).

(iii) Multi-indicator Standardization of Method Performance

To objectively compare the comprehensive performance of the method established in this study with those reported in existing literatures, it is necessary to standardize the core performance indicators (recovery rate, cost, time) to eliminate dimensional differences and achieve multi-dimensional comparability. According to the nature of the indicators, they are divided into two categories: positive indicators (recovery rate, %), where a higher value indicates better MPs recovery efficiency and accuracy; negative indicators (cost, U.S. dollar per sample; time, hour per sample), where a lower value indicates lower economic cost and higher operational efficiency. Standardization quantifies the original data into scores ranging from 0 to 100 through extreme value transformation:

Positive indicator (recovery rate)

Taking the maximum recovery rate among all methods as the benchmark, the calculation formula is as follows:

$$S_{\mathrm{recovery}}\;=\;\frac{X_{\max,\mathrm{recovery}}-X_{i,\mathrm{recovery}}}{X_{\max,\mathrm{recovery}}-X_{\min,\mathrm{recovery}}}\;\times \;100$$

where *X_i,recovery_* is the recovery rate of the *i*-th method, *X_max,recovery_* is the maximum recovery rate among all methods, and *X_min,recovery_* is the minimum recovery rate among all methods.

Negative indicators (time, cost)

Taking the minimum value of cost/time among all methods as the benchmark, the calculation formula is as follows:

$$S_{\mathrm{cost}/\mathrm{time}}=\frac{X_{\max,\mathrm{cost}/\mathrm{time}}-X_{i,\mathrm{cost}/\mathrm{time}}}{X_{\max,\mathrm{cost}/\mathrm{time}}-X_{\min,\mathrm{cost}/\mathrm{time}}}\times 100$$

where *X_i,cost/time_* is the cost/time of the *i*-th method, *X_max,cost/time_* is the maximum cost/longest time among all methods, and *X_min,cost/time_* is the minimum cost/shortest time among all methods. After standardization, a smaller score of all indicators indicates better performance (a small recovery rate score means the efficiency is close to the optimal; small cost and time scores mean better economy and efficiency). In the subsequent cost-performance radar chart, a closed triangle closer to the center indicates more excellent comprehensive performance, which facilitates the intuitive evaluation of method superiority.

#### 2.4.2. Statistical Methods

Pearson correlation analysis was applied to assess relationships between temperature and MPs particle size (normality was checked prior to parametric testing), with significance set at (*p* < 0.05). Time-dependent curves of soil mass loss and digestion efficiency under different temperatures were plotted to determine optimal conditions. For group comparisons, mean ± standard deviation (SD) were calculated, and Wilcoxon rank-sum tests were employed where data did not meet parametric assumptions. All analyses and visualizations were performed in R 4.3.1.

## 3. Results

### 3.1. Establishment of the Standardized Workflow

#### 3.1.1. Determination of Drying Parameters

(i) Drying Temperature

**Figure 2:**
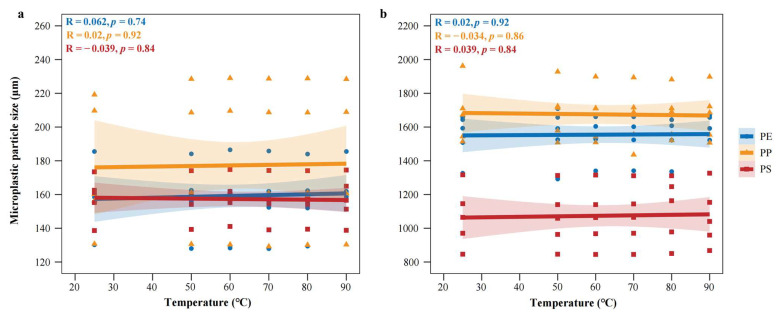
Pearson correlation analysis between drying temperature and particle size variation in MPs: (**a**) small-sized microplastics; (**b**) large-sized microplastics.

Across the tested temperature range of 25–90 °C, no significant differences were observed in the particle size of PE, PP, and PS MPs (Figure 2a,b).

**Figure 3:**
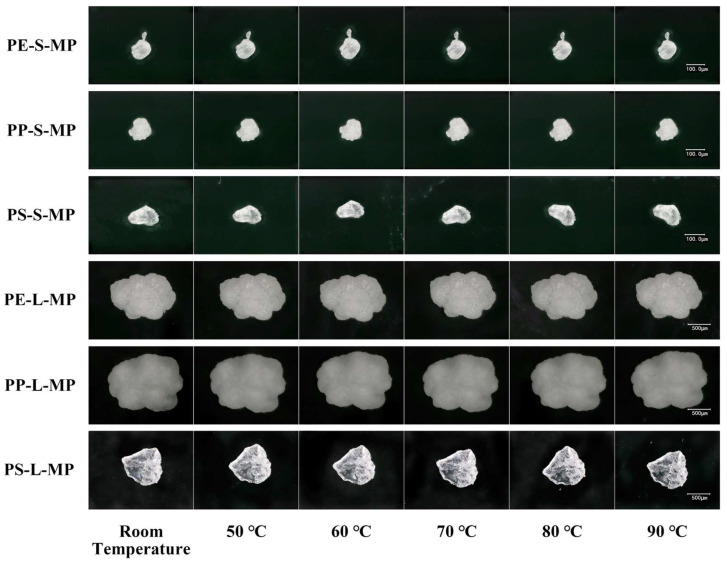
Effects of different drying temperatures on the morphology of MPs observed by a 3D ultra-depth microscopy system. PE-S-MP refers to small-sized polyethylene microplastics; PE-L-MP refers to large-sized polyethylene microplastics; PP-S-MP refers to small-sized polypropylene microplastics; PP-L-MP refers to large-sized polypropylene microplastics; PS-S-MP refers to small-sized polystyrene microplastics; PS-L-MP refers to large-sized polystyrene microplastics. All scale bars = 100 μm.

MPs maintained intact structure without melting, shrinkage, or cracking, and their surface morphology was indistinguishable at room temperature (Figure 3).

These results demonstrate that MPs are morphologically stable within this interval, validating it as a safe and practical temperature window for standardized drying procedures. Preliminary experiment results showed that LDPE exhibited obvious thermal deformation at 90 °C (Supplementary Figure S1). Consequently, 80 °C was finally selected as the treatment temperature.

(ii) Drying Time

**Figure 4:**
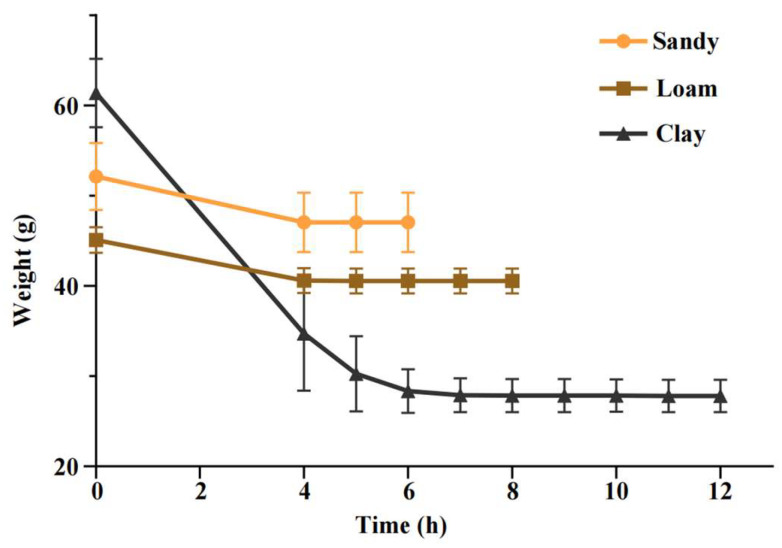
Drying curves of sandy, loam, and clay soils at 80 °C, showing the time required to reach constant weight.

The time required to reach constant weight varied among soil types: sandy soil stabilized after 5 h (47.05 ± 3.30 g), loam after 8 h (40.54 ± 1.38 g), and clay after 12 h (27.83 ± 1.82 g) (Figure 4). To eliminate variability and ensure complete drying across all soil textures, a standardized condition of oven-drying at 80 °C for 12 h was adopted for subsequent experiments.

#### 3.1.2. Determination of Density Separation Parameters

The density separation procedure was optimized through systematic evaluation of supernatant extraction method, container type, auxiliary separation, standing time, and number of separation cycles.

(i) Supernatant Extraction Method

**Figure 5:**
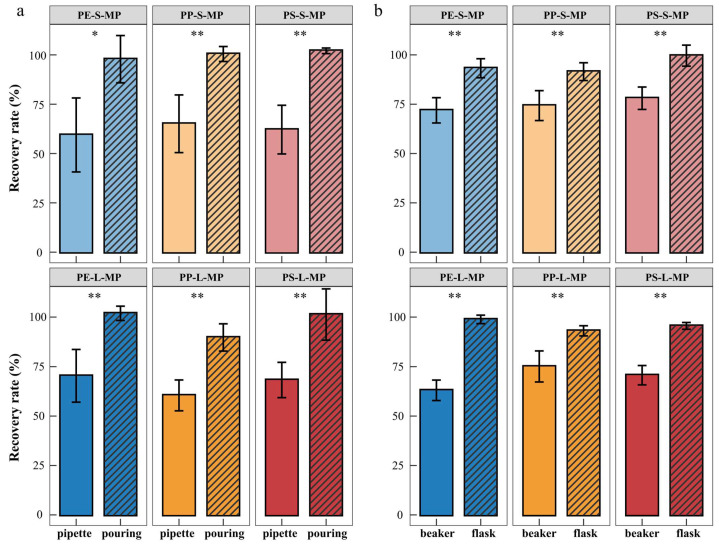
Effects of different supernatant extraction methods and containers on the recovery rate of MPs. (**a**) Comparison between decantation method and pipette method. (**b**) Comparison between Erlenmeyer flasks and beakers. PE-S-MP refers to small-sized polyethylene microplastics; PE-L-MP refers to large-sized polyethylene microplastics; PP-S-MP refers to small-sized polypropylene microplastics; PP-L-MP refers to large-sized polypropylene microplastics; PS-S-MP refers to small-sized polystyrene microplastics; PS-L-MP refers to large-sized polystyrene microplastics. Significance levels are denoted as * *p* < 0.05, ** *p* < 0.01.

The decantation method yielded consistently high recovery rates (89.70 ± 6.91% to 102.07 ± 1.41%), which were significantly greater than those obtained with the pipette method (59.42 ± 18.75% to 70.33 ± 13.29%). These results demonstrate that decantation provides a more efficient and stable recovery of MPs, and was, therefore, selected as the optimal supernatant extraction approach (Figure 5a).

(ii) Container Type

MP recovery was markedly higher when using Erlenmeyer flasks (91.51 ± 4.03% to 99.61 ± 4.77%) compared with beakers (63.04 ± 4.63% to 78.03 ± 5.08%). The conical geometry of Erlenmeyer flasks likely facilitated particle suspension and reduced losses during separation. Accordingly, Erlenmeyer flasks were adopted as the standard container for density separation (Figure 5b).

(iii) Auxiliary Separation Method

**Figure 6:**
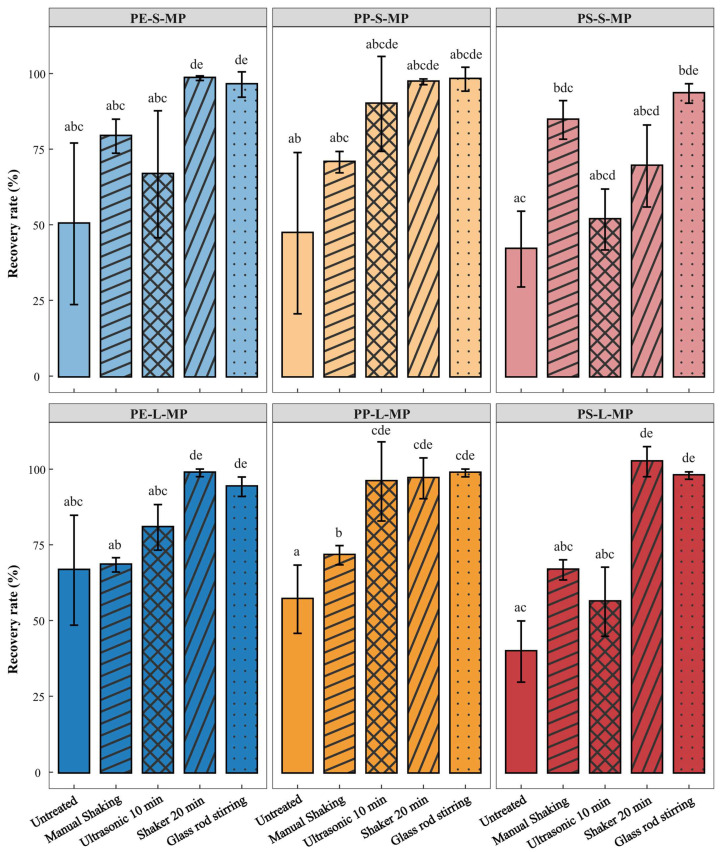
Effects of different auxiliary separation methods on the recovery rate of MPs. PE-S-MP refers small-sized polyethylene microplastics; PE-L-MP refers to large-sized polyethylene microplastics; PP-S-MP refers to small-sized polypropylene microplastics; PP-L-MP refers to large-sized polypropylene microplastics; PS-S-MP refers to small-sized polystyrene microplastics; PS-L-MP refers to large-sized polystyrene microplastics. Groups with the same lowercase letter indicate no significant difference (*p* > 0.05), while groups without common letters indicate significant differences (*p* < 0.05).

Different auxiliary separation methods had a significant impact on the recovery rate of MPs. Specifically, the recovery rate was low (39.76–66.60%) with high variability (SD: 10.09–26.72%) without any treatment; manual shaking showed limited improvement, with a maximum recovery rate of only 84.67%; 10 min of ultrasonication exhibited poor recovery efficiency for polystyrene (PS)-based MPs (PS-S-MP: 51.74 ± 10.07%); 20 min of shaking in a shaker resulted in high recovery rates for most MPs (96.99–102.49%), but the recovery rate of PS-S-MP remained low (69.43 ± 13.57%); glass rod stirring showed the optimal overall performance, as the recovery rates of all six types of MPs reached over 93.42% with small standard deviations and stable data, and it exhibited outstanding recovery efficiency for PS-S-MP was 93.42 ± 3.22%. Consequently, glass rod stirring was finally selected as the auxiliary separation method (Figure 6).

(iv) Settling Time

**Figure 7:**
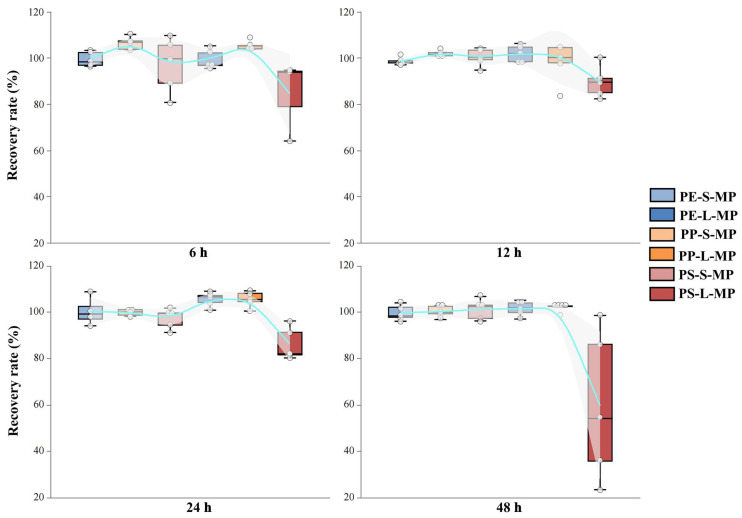
Effects of different settling times on the recovery rate of MPs. PE-S-MP refers to small-sized polyethylene microplastics; PE-L-MP refers to large-sized polyethylene microplastics; PP-S-MP refers to small-sized polypropylene microplastics; PP-L-MP refers to large-sized polypropylene microplastics; PS-S-MP refers to small-sized polystyrene microplastics; PS-L-MP refers to large-sized polystyrene microplastics.

Recovery rates stabilized after 12 h of natural sedimentation across all soil types (Figure 7). Extending the standing time beyond 12 h did not significantly improve recovery, indicating that 12 h is sufficient to achieve equilibrium in density separation. This duration was therefore adopted as the standard standing time.

(v) Density Separation Cycles

**Figure 8:**
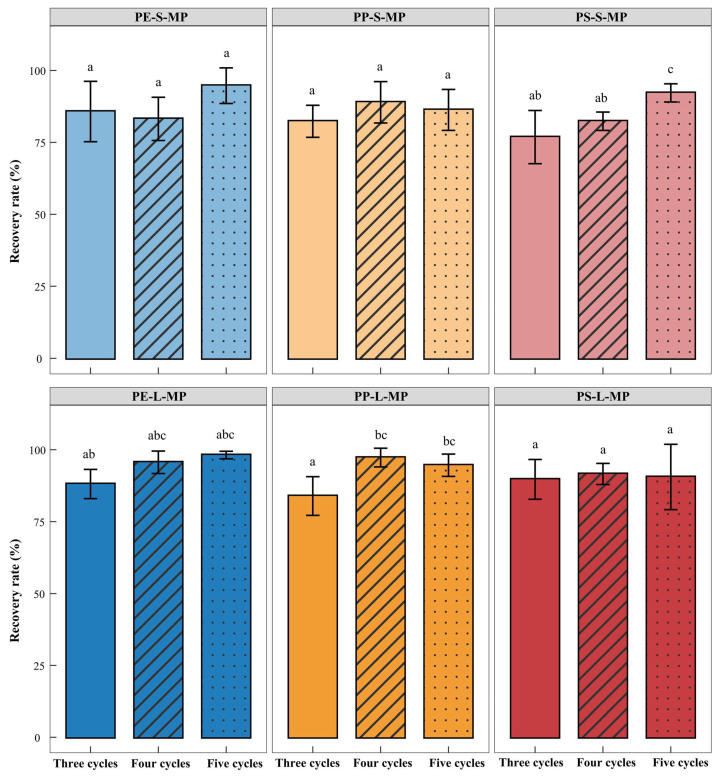
Effects of different density separation cycles on the recovery rate of MPs. PE-S-MP refers to small-sized polyethylene microplastics; PE-L-MP refers to large-sized polyethylene microplastics; PP-S-MP refers to small-sized polypropylene microplastics; PP-L-MP refers to large-sized polypropylene microplastics; PS-S-MP refers to small-sized polystyrene microplastics; PS-L-MP refers to large-sized polystyrene microplastics. Within the same group, groups with one or more identical lowercase letters show no significant difference (*p* > 0.05), while groups with no identical lowercase letters show significant differences (*p* < 0.05).

MP recovery increased with repeated separation cycles, but the improvement varied among polymers and particle sizes (Figure 8). For PE, the recovery rate rose steadily: PE-L-MP increased from 88.08 ± 5.08% (three cycles) to 98.16 ± 1.31% (five cycles), while PE-S-MP improved from 85.72 ± 10.49% to 94.70 ± 6.19%. For PP, PP-L-MP showed high recovery at four cycles (97.27 ± 3.25%) but slightly declined at five cycles (94.61 ± 3.88%), whereas PP-S-MP increased from 82.32 ± 5.56% (three cycles) to 88.93 ± 7.19% (four cycles), followed by a minor decrease at five cycles (86.28 ± 7.12%). For PS, recovery improved more consistently: PS-L-MP rose from 89.70 ± 6.91% (three cycles) to 91.58 ± 3.67% (four cycles) and remained stable thereafter (90.53 ± 11.35% at five cycles). PS-S-MP exhibited the most pronounced improvement, increasing from 76.83 ± 9.25% (three cycles) to 92.19 ± 3.16% (five cycles), which was 9.84% higher than at four cycles. Overall, considering both efficiency and stability, five separation cycles were determined as the optimal frequency for density separation.

#### 3.1.3. Determination of Digestion Parameters

(i) Digestion Temperature

**Figure 9:**
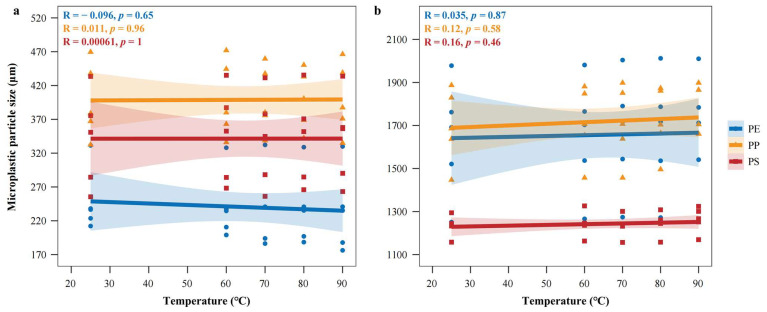
Pearson correlation analysis showing the relationship between digestion temperature and particle size change in microplastics: (**a**) small-sized microplastics; (**b**) large-sized microplastics.

**Figure 10:**
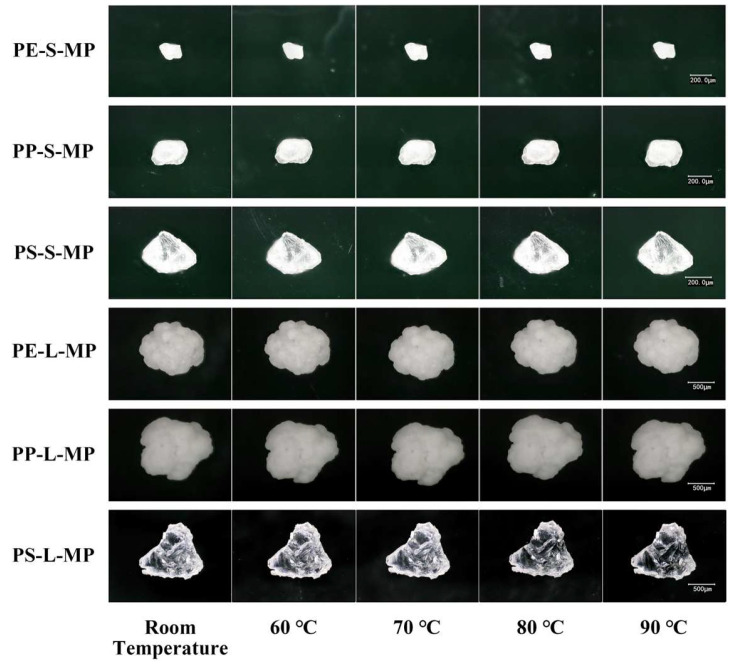
Morphological characteristics of MPs under different digestion temperatures.

The effect of temperature on MP integrity was assessed in the range of 60–90 °C. Within this interval, PE, PP, and PS MPs showed no significant changes in particle size (Figure 9), and ultra-depth 3D microscopy revealed intact structures with surface morphology consistent at room temperature (Figure 10). However, preliminary experiments indicated that LDPE began to deform at 90 °C (Supplementary Figure S1). Considering both recovery efficiency and particle preservation, 80 °C was selected as the optimal digestion temperature for subsequent experiments.

(ii) Volume Ratio of Supernatant to H_2_O_2_

**Figure 11:**
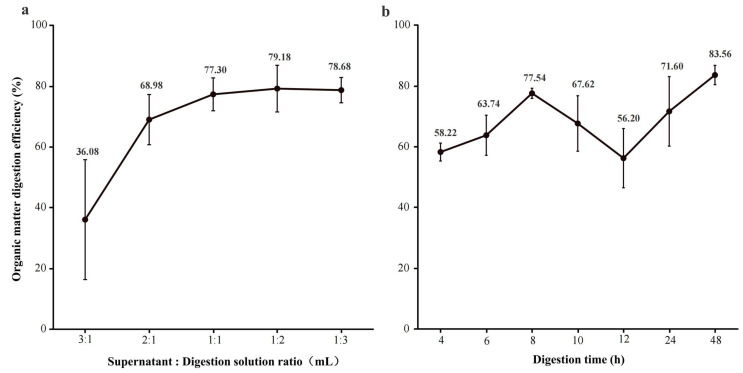
Effects of supernatant-to-digestion liquid ratio and digestion time on organic matter removal efficiency: (**a**) effect of volume ratio; (**b**) effect of digestion time.

The effect of varying the volume ratio of supernatant to 30% H_2_O_2_ on organic matter removal was assessed. At a ratio of 3:1, the removal rate was 36.08 ± 19.73%, whereas increasing the ratio from 1:1 to 1:3 improved removal to 77.30–79.18%. The highest efficiency (79.18 ± 7.68%) was achieved at a 1:2 ratio (Figure 11a). This ratio was, therefore, selected as optimal for the digestion step.

(iii) Digestion Time

Organic matter removal was evaluated over different digestion durations. At 4–6 h, removal rates ranged from 58.24% to 63.74%, reaching a maximum of 77.54 ± 1.67% at 8 h (Figure 11b). Extending the digestion to 12–48 h did not further improve efficiency and led to NaCl crystallization, which interfered with subsequent analysis. Consequently, 8 h was established as the optimal digestion time.

Finally, based on these optimization experiments, the digestion conditions were established as follows: a supernatant to 30% H_2_O_2_ volume ratio of 1:2, a digestion temperature of 80 °C, and a digestion duration of 8 h. These conditions were subsequently applied in the standardized workflow for soil MPs detection.

#### 3.1.4. Determination of Sequence for Density Separation and Digestion

**Figure 12:**
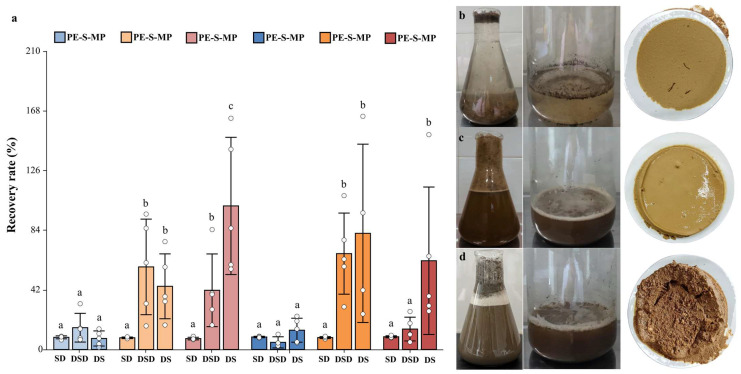
Effects of treatment sequences on organic matter distribution and MPs recovery rate, (**a**) comparison of recovery rates, (**b**) SD (density separation → digestion), (**c**) DSD (digestion → density separation → digestion), (**d**) DS (digestion → density separation). Within the same group, groups with one or more identical lowercase letters show no significant difference (*p* > 0.05), while groups with no identical lowercase letters show significant differences (*p* < 0.05).

The effect of different procedural sequences on MPs recovery was evaluated using spiked soil samples. Significant differences in recovery rates were observed among the three treatment sequences (Figure 12a). The SD group (density separation → digestion) showed recovery rates ranging from 76.83% to 89.70% (SD: 5.08–10.49%), with relatively stable results. In contrast, the DS group (digestion → density separation) exhibited recovery rates of 77.73–1010.86%, and the DSD group (digestion → density separation → digestion) ranged from 50.27–674.96%, both displaying high variability.

Visual observations showed that the DS and DSD groups generated bubbles during digestion; these bubbles entrapped soil particles, resulting in turbid supernatants and a large number of impurities remaining on the filter membranes (Figure 12b–d). Considering both recovery efficiency and data stability, the SD sequence (density separation → digestion) was finally selected as the standardized workflow for soil microplastic (MPs) detection.

#### 3.1.5. Determination of Quantitative Analysis Parameters

**Figure 13:**
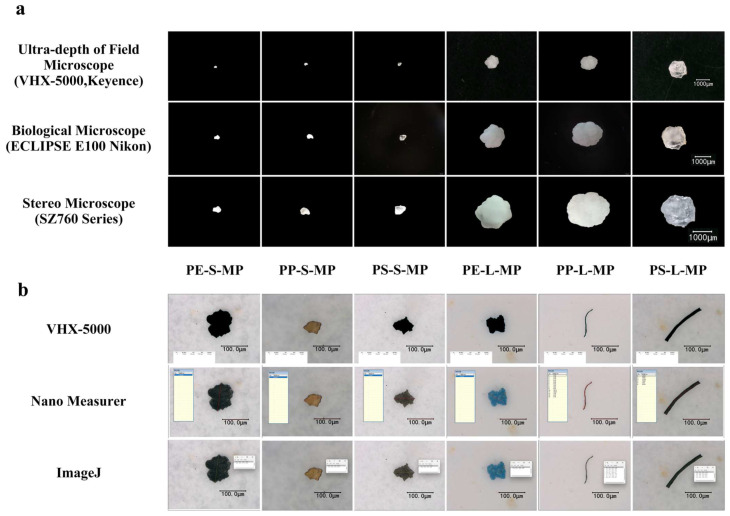
Comparison of microscopic observation and particle size analysis tools for MPs, (**a**) observation of MPs under different microscopes, (**b**) characterization of MPs by different particle size analysis software.

All three types of microscopes effectively revealed the morphological features of MPs within their respective magnification ranges. The stereomicroscope (10×–40×) is appropriate for particles from the millimeter scale down to several hundred micrometers, allowing clear observation of fibrous, flaky, and spherical forms, as well as surface scratches and wrinkles. Ultra-depth 3D microscopy, with its higher magnification capacity, further enables three-dimensional reconstruction of MP morphology (Figure 13a).

ImageJ software supports plugin expansion, allowing flexible setting of parameters such as threshold and edge detection to achieve particle size statistics. VHX-H2M software supports simultaneous measurement of multiple parameters, is compatible with various formats, and its functions are bound to the equipment. Nano Measurer requires manual scale calibration, has low efficiency in batch processing, and lacks customized analysis functions.

In this study, a stereomicroscope (equipped with an imaging system) combined with ImageJ software was used for the morphological observation and analysis of MPs (Figure 13b). The ImageJ analysis workflow was standardized as follows: scale was calibrated using a stage micrometer; a uniform grayscale threshold (0–80) was applied for binarization; the “Analyze Particles” function was used with a size limit of >100 μm (corresponding to a diameter of ~100 μm) to exclude smaller debris; touching particles were manually separated using the wand tool or by threshold adjustment before counting. All analyses were conducted by a single operator to ensure consistency.

### 3.2. Adaptability Verification of the Standardized Protocol

**Figure 14:**
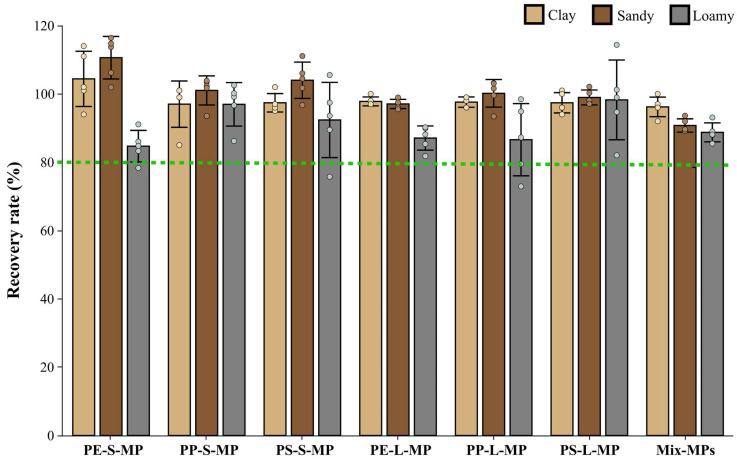
Recovery rates of the standardized MPs detection method established in this study in sandy soil, loam, and clay. The green dotted line indicates a recovery rate of 80%.

The MPs extraction method established in this study exhibited efficient extraction capability in sandy soil, loam soil, and clay soil, with an overall average recovery rate of 96.43% (Figure 14). In the sandy soil group, the recovery rate of Mix-MPs was 96.20 ± 2.86%, and the recovery rates of single type MPs ranged from 97.00% to 104.40%; in the loam soil group, the recovery rate of Mix-MPs was 90.75 ± 1.96%, and the recovery rates of single-type MPs ranged from 97.06% to 110.61%; in the clay soil group, the recovery rate of Mix-MPs was 88.74 ± 2.78%, and the recovery rates of single type MPs ranged from 84.70% to 98.25%. This method can achieve stable and efficient recovery of MPs in different types of soils.

### 3.3. Complete Procedure for Soil Microplastic Detection

**Figure 15:**
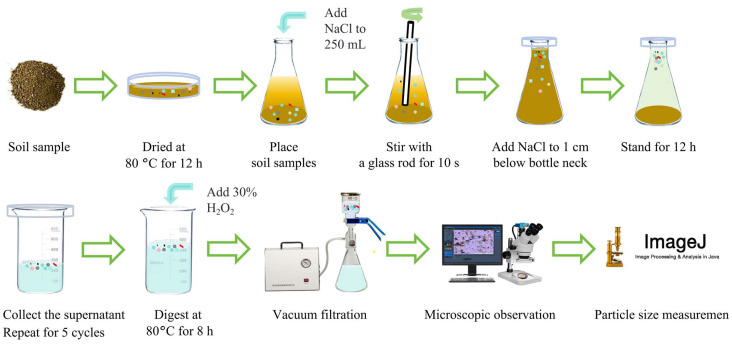
Flow chart of the standardized experimental process.

Based on the optimized parameters and standardized workflow established in this study, a complete procedure for the quantitative detection of MPs in soil was formulated, as detailed in Table 2 and Figure 15.

### 3.4. Cost-Effectiveness Comparison of the Entire Process

**Figure 16:**
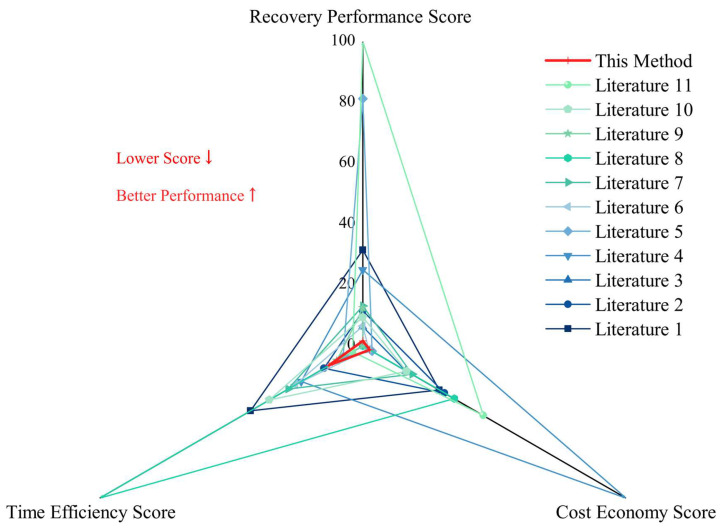
Comparison of performance indicators among different MPs detection methods. A smaller score indicates better performance of the corresponding indicator.

The standardized soil MPs detection method developed in this study demonstrated clear advantages in three key aspects: recovery rate, cost, and processing time (Figure 16). The median recovery rate reached 97.2%, exceeding the median recovery rate of 92% reported in recent literature. Economically, the processing cost per sample was only USD 9.77, substantially lower than the literature average of approximately USD 49.05. In terms of operational efficiency, the total duration of the complete procedure was 68 h, compared with an average of 113.81 h reported in previous studies, representing a marked improvement in analysis throughput.

Overall, this method maintains a high recovery rate while significantly reducing cost and processing time. By balancing accuracy, economy, and efficiency, it provides a practical and standardized technical solution for quantitative assessment of MPs in soil.

## 4. Discussion

### 4.1. Analysis of Key Parameters in the Standard Procedure

By verifying four key steps—drying, density separation, digestion, and quantification—and systematically optimizing the parameters for each, this study established a standardized procedure for detecting MPs in soil. The procedure is characterized by high recovery rates, good repeatability, low cost, and short processing time, providing a scientific and technical basis for standardizing MP quantification in soil.

Drying is the primary step in the quantitative detection of MPs in soil, which needs to ensure the intact morphology of MPs and complete removal of soil moisture. Previous studies typically dried soil samples at 40–60 °C to prevent MP deformation [43,44,45,46,47], which required 24–72 h for complete moisture removal [44,46,48,49]. In contrast, drying at 80 °C for 12 h in this study preserved the morphology of PE, PP, and PS MPs, consistent with [50], and allowed soils of various textures to reach constant weight efficiently. Meanwhile, sandy soil, loam soil, and clay soil all reached constant weight after 12 h of drying at 80 °C, which is in agreement with the study by O’Kelly et al. (2005) [51]. Therefore, drying at 80 °C for 12 h can significantly shorten the processing time and improve experimental efficiency while ensuring the integrity of MPs and the effectiveness of soil drying.

Density separation is the second step in the quantitative detection of MPs. Currently, most studies do not describe the specific operation of supernatant extraction in detail; the method section usually only mentions ‘transferring the supernatant’ without specifying details [52,53,54]. A few studies have referred to the use of decantation or pipetting for supernatant extraction [55,56,57]. In this study, comparative experiments showed that the recovery rate of the pipetting method was significantly lower than that of the decantation method, with a loss of 30.01%, and the decantation method had better data stability (its standard deviation was 6.1% lower than that of the pipetting method). This result is consistent with the observation by Gran et al. (2025) that the decantation method performed better in multiple salt systems [58]. The decantation method enables continuous transfer of the surface enriched zone through the stable decline of the liquid level, which is conducive to the efficient recovery of MPs. In contrast, the pipetting method easily causes liquid adhesion to the tube wall, leading to MPs being retained with the residual liquid and resulting in recovery loss. Experimental comparison showed that Erlenmeyer flasks achieved significantly higher MP recovery than beakers. This is likely because their narrow mouths reduce contact area between the liquid and the container wall, minimizing MP adsorption. In contrast, Erlenmeyer flasks have a narrow mouth, which reduces the liquid-wall contact area and adsorption probability, thereby minimizing loss. In conclusion, using Erlenmeyer flasks for supernatant decantation can effectively improve the recovery rate of MPs and data stability, and has good operability and application value for standardization.

Mechanical auxiliary treatment is a key step in density separation, aiming to break down soil aggregates, release encapsulated MPs, and improve the recovery rate [59]. However, there is currently a lack of unified standards for this operation, and some studies do not perform any auxiliary treatment after extracting the supernatant [60,61,62]. The results of this study showed that the recovery rate of MPs in the group without auxiliary treatment was only 39.76–66.60%, indicating that insufficient mechanical disturbance would lead to inadequate release of MPs, significantly affecting the detection accuracy. Currently commonly used methods include ultrasonic treatment, shaker oscillation, manual shaking, and glass rod stirring [63,64,65,66,67]. This study found that ultrasonic treatment and shaker oscillation have limitations: the recovery rate of PS MPs was 69.43 ± 13.57% after 20 min of shaker oscillation; after 20 min of ultrasonic treatment, the recovery rates of all three types of MPs decreased significantly. This likely resulted from high-intensity disturbance causing MPs to aggregate with impurities, increasing their adsorption on the container walls [68]. In contrast, manual shaking and glass rod stirring are easy to operate and do not require special equipment, making them more suitable for low-cost scenarios. Among them, the recovery rate of glass rod stirring was significantly higher than that of manual shaking, because it allows manual control of force and frequency to achieve gentle and uniform disturbance. This not only effectively breaks down aggregates but also reduces re-adsorption and loss on the container wall. In conclusion, selecting glass rod stirring as the auxiliary method can ensure high recovery rate and stability while reducing equipment dependence and cost, thus having good potential for standardized application.

After completing the mechanical auxiliary treatment, static flotation requires a certain period of time to separate impurity sedimentation from MP flotation. Most current studies use a static time of 24–48 h [52,53,69]. This study found that MPs had fully floated and the recovery rate reached the maximum when static for 12 h; when the static time was extended to 24 or 48 h, the recovery rate decreased significantly. This result is consistent with the observation by Maisto et al. (2022) [70]. The observed decrease in recovery with extended static time is likely due to aggregation of floated MPs with incompletely settled fine particles, forming higher density complexes that re-sediment [70]. Therefore, a 12 h static period ensures efficient separation while minimizing processing time.

To improve the recovery rate of MPs, most current studies often adopt a strategy of repeated density separation, gradually enriching MPs through multiple rounds of flotation [23,64,71]. Existing methods mostly perform three cycles using high-density salt solutions such as ZnCl_2_ and NaI [52,56,69,72]. Although these methods have high separation efficiency, they have problems such as high cost, complex waste liquid treatment, and high toxicity, which easily cause environmental pollution and limit their application under resource limited conditions [73]. In contrast, NaCl is non-toxic, cheap, and easily available, which is more in line with the principles of green chemistry and has significant advantages in sustainability and practicality [74]. This study investigated the effect of the number of density separation cycles on the recovery rate based on NaCl medium. The results showed that increasing the number of cycles could significantly improve the recovery rate. Although five cycles increased the time consumption, the recovery rate reached 86.28–98.16%, and there was no need to use highly toxic and high-cost reagents.

Digestion is the third step in the quantitative detection of MPs, aiming to remove organic matter and reduce its interference with subsequent identification and quantification. Key parameters include digestion temperature, volume ratio of supernatant to H_2_O_2_, and digestion time, which collectively affect the morphological integrity of MPs and the efficiency of organic matter removal. Most current studies use 50–70 °C to avoid MP deformation [52,53,64,71]. In this study, after treatment with H_2_O_2_ at 80 °C, PE, PP, and PS MPs remained intact without melting or cracking, which is consistent with the report by [75] that PE and PP showed good stability in the Fenton system at 70 °C. Secondly, most existing studies have not clearly specified the specific value of the volume ratio of supernatant to H_2_O_2_ [64,65,71,76]. Only M. Zhang et al. (2023) adopted a ratio of 10:1 (supernatant: 30% H_2_O_2_) in their study on the distribution of MPs in loess plateau soils [77]. In this study, when testing different ratios at 80 °C with 12 h of standing, it was found that when the volume ratio increased from 1:1 to 1:3, the organic matter removal rate reached 77.30–79.18%; among them, the ratio of 1:2 had the optimal effect, ensuring efficient removal of non-target substances while reducing reagent consumption and lowering costs. After determining 80 °C and the ratio of 1:2, the time was further optimized. Most current methods require more than 24 h [37,49,52,71], while this study found that 8 h could achieve a removal rate of 77.54 ± 1.67%; extending to 24 h caused severe salting-out due to water evaporation, leading to crystal adhesion to the container wall, destroying solution homogeneity, and interfering with subsequent operations. In summary, the combination of 80 °C, supernatant: H_2_O_2_ = 1:2, and 8 h of digestion can efficiently remove organic matter while ensuring the morphological integrity of MPs, significantly shortening the time, saving reagents, avoiding salting-out problems, and having both efficiency, stability, and economy. It is worth noting that the ~79% organic matter removal rate is high-efficiency under the premise of protecting MP morphology—harsher conditions for complete digestion would cause deformation of MPs. Nevertheless, residual organic matter may interfere with visual MP identification: fragmented fibers and humic aggregates may be misclassified as small-sized MPs, and adsorbed organic matter may mask MPs’ typical surface features. This interference is acceptable for baseline monitoring; supplementary purification steps (e.g., secondary density separation) can be applied for high-precision analysis.

In the quantitative detection of MPs in soil, the sequence of density separation and digestion directly affects experimental efficiency and result accuracy. Some current studies adopt the ‘digestion followed by separation’ (DS) or ‘digestion–separation–redigestion’ (DSD) procedures, which aim to enhance organic matter removal through early or repeated digestion. This study identified clear drawbacks in the DS and DSD approaches: the direct reaction of soil samples with high-concentration H_2_O_2_ produces substantial oxygen bubbles, often leading to solution overflow and filter membrane clogging by soil particles, thereby prolonging suction filtration time by 3–5 fold. Moreover, the DS procedure generally requires 24–72 h of digestion [62,78,79], while the DSD process entails even longer total duration due to secondary digestion and multiple transfer steps [80], both are operationally complex and increase the risk of MP loss. By contrast, the ‘density separation followed by digestion’ (SD) procedure effectively avoids these issues. First, density separation removes most inorganic particles, which reduces the digestion burden, decreases H_2_O_2_ consumption, and results in a gentler reaction without vigorous bubbling. This prevents filter membrane blockage and ensures smooth suction filtration. Digestion under this scheme requires only 8 h, cutting total processing time by more than 50% compared to the DS and DSD workflows. Furthermore, fewer transfer steps minimize physical loss of MPs and help improve recovery rates.

Quantitative analysis is the fourth step in the detection of MPs in soil, with core objectives of identification, counting, and particle size measurement. Its cost and efficiency depend heavily on the choice of instruments and software. Currently, most studies rely on high-end spectroscopic and thermal techniques, such as Fourier transform infrared spectroscopy (FTIR), Raman spectroscopy, pyrolysis-gas chromatography/mass spectrometry (Py-GC/MS), or thermogravimetric analysis (TGA), which enable precise polymer identification [71,81,82]. However, these instruments are expensive, complex to operate, and costly to maintain, limiting their use in resource-constrained laboratories. In this study, we employed a stereomicroscope combined with an external display for analysis, allowing clear observation of MP morphology to meet basic identification and counting needs [59,83]. The instrument’s purchase cost is only 1/16 to 1/50 that of an FTIR system, and it is easier to operate and maintain, making it more accessible for laboratories with limited resources [84]. After comparing several image analysis tools, we selected ImageJ as the optimal software. It is open-source, free, has low hardware requirements, and offers intuitive core functions such as particle counting and size measurement [33,85]. Its widespread international use and strong compatibility also facilitate method promotion and data sharing. Unlike spectroscopic and thermal techniques, this protocol system focuses on high-throughput morphological screening. It does not provide polymer-specific identification (e.g., distinguishing PE from PP) and cannot chemically differentiate MPs from natural particles. When detailed compositional confirmation is needed, these limitations can be addressed by supplementing with portable spectroscopic tools or targeted digestion treatments. In summary, this protocol offers a low-cost, efficient, and universally accessible approach for MP quantification. It avoids the barriers associated with high-end equipment and complements more precise analytical techniques, providing an economical and practical solution for grassroots laboratory analysis.

### 4.2. Method Evaluation and Research Significance

The quantitative detection procedure for MPs in soil established in this study exhibits favorable performance in terms of recovery rate (covering different soil types, MP species, and particle sizes), time efficiency, and cost control, providing reliable support for the method as a standardized baseline approach.

The MP detection procedure established in this study shows good applicability and high recovery rates in three typical textured soils: sandy soil, loam soil, and clay soil. Most existing studies focus on method verification and optimization in sandy soil and loam soil matrices [86,87], while systematic evaluation of MP recovery efficiency in clay soil is relatively scarce. This is closely related to the constraints imposed by the unique physicochemical properties of clay on MP extraction. Zhang et al. (2018) pointed out in their study that clay has high clay particle content, strong aggregation, and surface adsorption properties [88]. MPs are easily encapsulated inside soil aggregates and can form stable complexes with clay particles through physical adsorption, making it difficult to release them from the soil matrix. This places higher demands on the separation efficiency of detection methods. Le et al. (2025) also indicated that high concentrations of Zn^2+^ may neutralize surface charges, promoting ‘clay-MP’ aggregation [89]; residual organic matter interferes with fluorescence identification; and the strong aggregate structure hinders MP release, especially affecting small-sized particles. They reported that the recovery rate of low-density polyethylene (LDPE) in clay soil was only 25 ± 11%. In this study, by optimizing mechanical assistance, multiple rounds of density separation, and treatment sequence, the release efficiency of MPs in clay soil was improved. Experimental results showed that the overall average recovery rate in clay soil reached 88.74 ± 2.78%, with stable recovery of all MP types: large-sized PS was 98.25 ± 11.68%, small sized PS was 92.36 ± 11.01%; small sized PP was 96.95 ± 6.37%; small sized PE was 84.70 ± 4.62%, and large sized PE was 87.08 ± 3.54%. This method can achieve efficient and stable recovery in all three soil types without adjusting core parameters according to soil texture, demonstrating good matrix universality and providing technical support for standardized detection and cross-study data comparability.

The method established in this study exhibits good adaptability and high recovery rates for three common MP types in soil: PE, PP, and PS, effectively meeting the quantitative needs of major MP components in actual samples. Existing studies show that different methods have varying recovery effects on these three MP types, and some studies have also pointed out recovery limitations for specific types in their own verification. For example, Han et al. (2019) found in the verification of their density separation method that the recovery rate of PE was 78 ± 16%, the lowest among the 6 MP types tested, and the standard deviation (16%) was significantly higher than that of PS (4%) [86]. The study suggested that the white appearance of PE is similar to that of inorganic particles such as quartz and feldspar, making it prone to missed detection in visual identification, leading to low recovery rates and data fluctuations. Similarly, Radford et al. (2021) reported a PP recovery rate of only 51 ± 11% in their extraction method, the lowest among the 12 MP morphologies tested [78]. This was attributed to the high flexibility and large specific surface area of PP fibers, which easily entangle with soil particles or organic residues and are difficult to float effectively in rapeseed oil flotation. In contrast, the present method achieves efficient and stable recovery for all three MP types: PE ranges from 92.1% to 98.16%, PP ranges from 89.5% to 96.3%, and PS ranges from 86.28% to 94.7%. The recovery rate of each type is close to or higher than 90% with small data fluctuations. These results indicate that the method has good adaptability to different MP types, effectively coping with the complex situation of coexisting multi-component MPs in soil and providing reliable support for accurate quantification.

The method established in this study demonstrates good adaptability and high recovery rates for MPs of different particle sizes in soil. Current mainstream extraction methods face two primary limitations regarding particle size adaptability. First, some methods have a limited coverage range, focusing only on specific sizes. For example, G. Li et al. (2024) reported that their Plastic Flotation Separation System (PFSS) efficiently recovers small PS (45 μm) and PET (40 μm) particles (<60 μm) with 90% recovery [90]. However, they noted that systematic verification for larger particles (>100 μm) commonly found in soils has not been conducted, indicating a need for broader validation to fully assess MP size distributions in soil. Second, even methods covering multiple size ranges often show inconsistent recovery across sizes. Radford et al. (2021) in comparing three density separation methods (NaCl, ZnCl_2_, and rapeseed oil), tested MPs of various morphologies and sizes (0.25–0.5 mm, 0.5–1 mm, and 1–5 mm) [78]. Their data revealed that even the best-performing rapeseed oil method recovered small PVC (0.25–0.5 mm) at 99%, while recovery of PP fibers (1–5 mm) was only 78%, highlighting variability that can affect cross-size data comparability. In contrast, the present method achieves efficient and stable recovery across both small (127.9–473.7 μm) and large (1251–2012 μm) particle size ranges, with average recoveries of 95.47% (SD 4.98%) and 98.72% (SD 8.21%), respectively, and low variation. This demonstrates consistent performance across a wide size spectrum, supporting its use as a baseline method for multi-size MP detection in soil. Accordingly, this protocol is specifically designed and validated for MPs larger than approximately 100 μm, with an effective detection limit near this threshold due to the resolution constraints of stereomicroscopy. Consequently, the inability to quantify MPs smaller than 100 μm is a defined limitation of the method.

At the technical promotion level, cost and efficiency are key factors affecting the application of the method in grassroots laboratories. Le et al. (2025) mentioned in their study that although they achieved a recovery rate of > 90% for MPs of 500–1000 μm [89], the method relies on fluorescence microscopes, high purity reagents, and special consumables, resulting in high overall detection costs, which poses a challenge for laboratories with limited resources. Scopetani et al. (2020) also pointed out that although their olive oil-based method is environmentally friendly, the unit price of key consumables (such as hydrophobic filter membranes) is high, and it requires supporting special oscillation equipment [91]. The equipment threshold limits its wide application. In response to this, the present study uses cheap and easily available sodium chloride (NaCl) as the flotation medium (unit price approximately 1/50 that of sodium iodide and 1/20 that of zinc chloride), avoids the use of special equipment such as ultrasonic instruments and constant-temperature shakers, and only requires conventional utensils such as Erlenmeyer flasks and glass rods throughout the process, significantly reducing instrument and operation costs. The detection cost per sample is controlled at USD 9.77, demonstrating good economic feasibility. Meanwhile, existing methods often take too long due to complex procedures. Katsumi et al. (2022) pointed out in their study that although their method has a recovery rate of > 90% for MPs of 20–150 μm and low equipment dependence, it includes multiple centrifugations, three rounds of 24 h digestion, and long term standing, with a total time consumption exceeding 100 h, making it difficult to meet the needs of batch detection [92]. Through process optimization, this study shortened the total time consumption to 68 h; the density separation step is easy to operate, requiring only glass rod stirring, Erlenmeyer flask decantation, and 12 h standing without special equipment, and supports simultaneous processing of 60 samples, greatly improving throughput. This design balances cost, efficiency, and operability, providing a practical and promotable technical path for grassroots laboratories in developing countries to conduct quantitative analysis of MPs in soil.

The standardized, cost-effective, and widely accessible detection protocol presented here extends its value beyond technical analysis by providing a foundational data basis for environmental risk assessment and policy development as a baseline monitoring tool. The accurate quantitative data it generates provide a critical foundation for evaluating the ecological risks of MPs to soil biota and soil health, as well as for assessing potential human exposure routes (e.g., via the food chain) [49]. By enabling reproducible and large-scale monitoring, this approach can contribute to the establishment of science-based regulatory frameworks and targeted mitigation strategies [47].

### 4.3. Future Directions and Expansions of the Method

Despite the method’s strengths in standardization, cost-effectiveness, and adaptability to different soils, there is room for further improvement. First, to address morphological identification bias, future work can pair this workflow with spectroscopic techniques like µFTIR or Raman spectroscopy. This confirms polymer types and reduces misclassification of non-plastic particles. Second, inter-laboratory validation with multiple teams and regional soils will test reproducibility across different settings. This helps promote the method as a widely accepted baseline standard, especially for resource-limited regions. Third, we can optimize recovery of high-density polymers (e.g., PVC, PET) by using high-density reagents such as ZnCl_2_ or modified flotation strategies, while retaining low costs. Additionally, expanding the method to sediment, irrigation water, or atmospheric deposition enables cross-media MPs monitoring. This provides a more holistic understanding of their migration in terrestrial ecosystems. These extensions will strengthen the method’s rigor and practical value, supporting harmonized global soil MPs assessment.

## 5. Conclusions

This study established a standardized, reproducible, and cost-effective method for the quantitative detection of MPs in soil. The procedure involves drying soil samples at 80 °C for 12 h, followed by density separation using Erlenmeyer flasks. The supernatant is collected via decantation, combined with 10 s of glass rod stirring and 12 h of static settling, and repeated five times. Digestion is performed at 80 °C for 8 h with a hydrogen peroxide (H_2_O_2_) to supernatant volume ratio of 1:2. Finally, MPs are quantitatively analyzed using a stereomicroscope coupled with ImageJ software. This method achieved an average spiked recovery rate of 96.43%, with a total processing time of 68 h and a per-sample cost of USD 9.77, demonstrating high efficiency, accuracy, and affordability. This protocol has inherent limitations: sodium chloride (NaCl) cannot recover high-density polymers, the detection threshold is limited to particles > 127 μm, and polymer characterization requires supplementary spectroscopy.

The standardized procedure enhances data comparability across studies and provides a practical, low-cost technical pathway for MPs monitoring, particularly in resource limited laboratories. Looking forward, this method can serve as a foundation for expanding MPs detection to other environmental matrices, including water and air, facilitating the establishment of unified quantitative standards. Such efforts will support coordinated cross-media monitoring, improve global understanding of MPs transport and environmental risks, inform transnational pollution control policies, and contribute to the achievement of the United Nations 2030 Sustainable Development Goals.

### Environmental Implication

MPs are widely present in soil environments and have become a critical environmental issue threatening the stability of soil ecosystems and human health. They not only disrupt soil physicochemical properties and biogeochemical cycles but also may accumulate through the food chain, exerting potential toxic effects on plants, animals, and humans. Therefore, efficient and accurate extraction and detection of MPs from complex soil samples are of crucial significance for assessing soil MPs pollution levels and clarifying their environmental fates. This study reports an efficient and low-cost standardized method for detecting MPs in soil. This method possesses both high recovery rate and practicality. It not only features low processing cost and short analysis cycle but also is applicable to different types of soil samples such as sandy soil, loam, and clay. It provides reliable technical support for large-scale survey and monitoring of MPs in soil environments and assessment of their pollution risks.

## Figures and Tables

**Table 1:** Specific treatment methods for density separation and digestion sequences.

Treatment Group	Key Operational Steps
Density separation → Digestion (SD)	Perform 3 flotation cycles under optimized conditions (standing for 12 h) → combine supernatants + 50 mL 30% H_2_O_2_ → digest at optimal temperature for 12 h → suction filtration through 0.45 μm filter membrane → dry to constant weight.
Digestion → Density separation → Digestion (DSD)	Initial digestion: sample + 50 mL 30% H_2_O_2_ (24 h at room temperature) → 3 flotation cycles → combine supernatants + 50 mL 30% H_2_O_2_ → digest at optimal temperature for 12 h → suction filtration and drying.
Digestion → Density separation (DS)	Sample + 50 mL 30% H_2_O_2_ (24 h at room temperature) → 3 flotation cycles → combine supernatants → suction filtration → dry to constant weight at 80 °C.

Note: Abbreviation explanation, S meaning density separation step, D meaning digestion step.

**Table 2:** Complete procedure for soil microplastic detection.

Step	Procedure	Key Parameters
Sample Pretreatment and Drying	1. Sieve soil sample through a 5 mm stainless steel mesh; 2. Transfer 50 mL homogenized sample to a glass petri dish; 3. Dry in a constant-temperature blast drying oven.	Temperature: 80 °C Duration: 12 h
Density Separation	1. Transfer dried soil to a 250 mL conical flask; 2. Add saturated NaCl solution to 250 mL mark, then add fresh saturated sodium chloride solution to 1 cm below the rim of the conical flask; 3. Stir with glass rod (10 s), rinse rod with DI water; 4. Allow to settle for 12 h; 5. Decant ~50 mL supernatant into 800 mL beaker; repeat 5 times.	Solution: Saturated NaCl (density ~1.20 g/cm^3^) Settling time: 12 h per cycle Total supernatant collected: ~250 mL
Organic Matter Digestion	1. Combine all supernatants (~250 mL); 2. Add 30% (*w*/*w*) H_2_O_2_ at a volume ratio of 1:2 (supernatant: H_2_O_2_); 3. Incubate in oven at 80 °C for 8 h; cool to room temperature.	Oxidizing agent: 30% H_2_O_2_ Ratio: 1 part supernatant: 2 parts H_2_O_2_ Temperature: 80 °C Duration: 8 h
Microplastic Collection and Quantification	1. Vacuum-filter the digested solution through a 0.45 μm membrane (47 mm diameter); 2. Place filter on clean plate; 3. Observe under stereomicroscope with digital imaging; 4. Analyze images using Image J software.	Filter type: 0.45 μm pore size, 47 mm diameter Instrument: Stereomicroscope + digital camera Software: Image J
Quality Control	1. Rinse all glassware 3× with 0.45 μm-filtered deionized water; 2. Wear nitrile gloves throughout; 3. Run process blanks (reagent-only) and lab environment blanks per batch.	Blank types: Process blank and environment blank Contamination control: Minimize plastic use; use non-plastic tools where possible

## Data Availability

Data will be made available on request.

## Supplementary Materials

The following supporting information can be downloaded at: https://www.mdpi.com/article/10.3390/toxics14010105/s1. Figure S1. Morphological Characterization of Self-Prepared MPs Under Different Drying Temperatures. Figure S2. Comparison of Microplastic Recovery Rates Under Different Treatment Durations of Ultrasound and Shaker. Table S1. Summary of Key Experimental Parameters and Reporting Completeness in Soil Microplastics Detection Studies (2020–2024). Table S2. Comparison of Cost, Time Consumption and Recovery Rate Among Different Soil Microplastics Detection Methods. References [23,44,46,47,48,49,52,53,54,55,56,57,60,61,62,63,64,65,66,67,69,71,72,76,77,78,79,80,81,82,86,87,89,91,92,93,94,95,96,97,98,99,100,101,102,103,104,105,106,107,108,109,110,111,112,113,114,115,116,117,118,119,120,121,122,123,124,125,126,127,128,129,130,131,132,133,134,135,136,137,138,139] are cited in the Supplementary Materials.
