# Supplementary material for: A Cost-Effective Standardized Quantitative Detection Method for Soil Microplastics in Different Substrates
Source: Toxics. 2026 Jan 22;14(1):105. doi: 10.3390/toxics14010105 (PMC12845626; doi:10.3390/toxics14010105)
Supplement: Supplementary file 1 [file toxics-14-00105-s001.zip › Supplementary Figures.pdf]

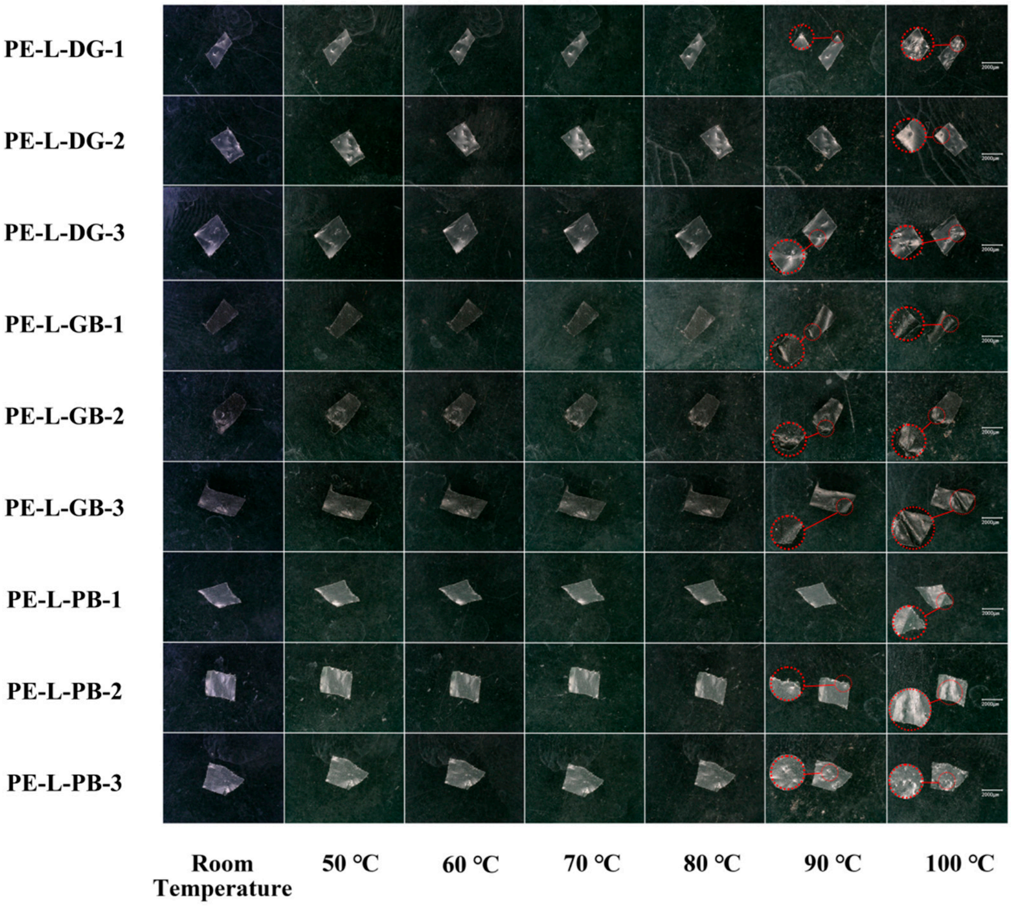

Figure S1. Morphological Characterization of Self-Prepared MPs Under Different Drying Temperatures.

Note: DG stands for Disposable Gloves; GB stands for Garbage Bag; PB stands for Plastic Bag.

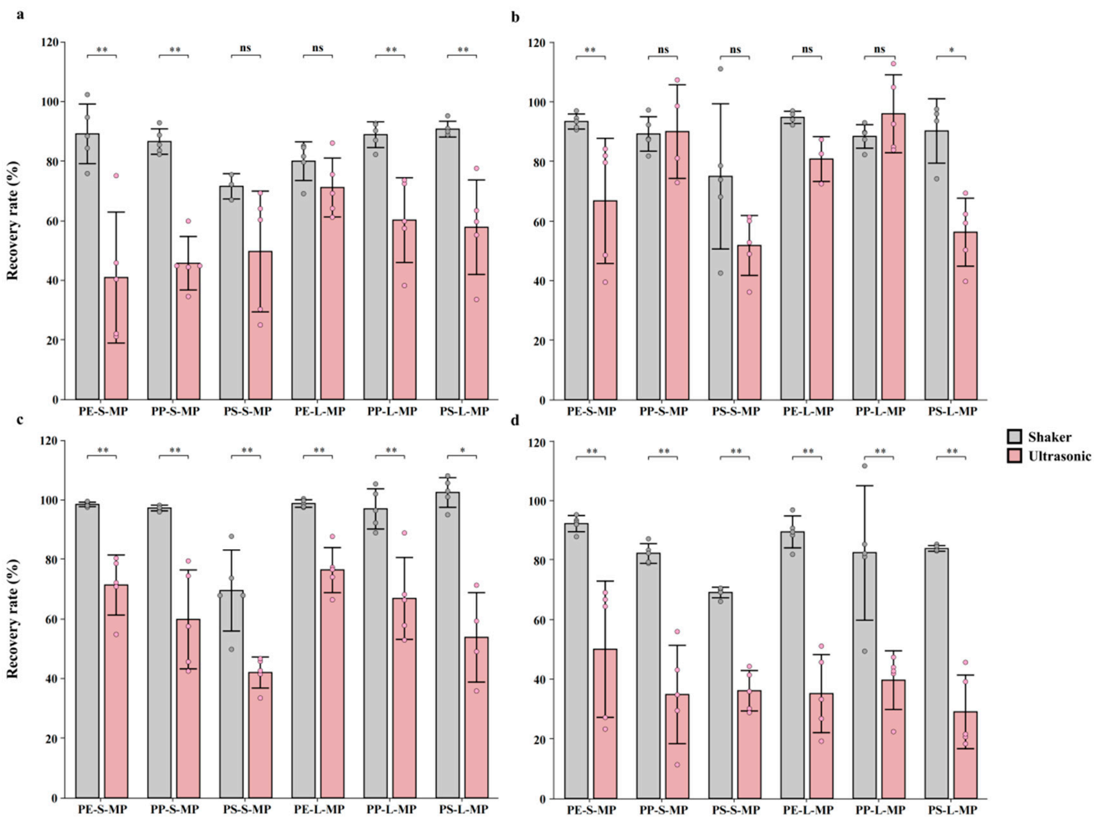

Figure S2. Comparison of Microplastic Recovery Rates Under Different Treatment Durations of Ultrasound and Shaker. (a) 5 min; (b) 10 min; (c) 20 min; (d) 30 min. PE-S-MP refers small-sized polyethylene microplastics; PE-L-MP refers large-sized polyethylene microplastics; PP-S-MP refers small-sized polypropylene microplastics; PP-L-MP refers large-sized polypropylene microplastics; PS-S-MP refers small-sized polystyrene microplastics PS-L-MP refers large-sized polystyrene microplastics. Significance levels are denoted as *P < 0.05, **P < 0.01 and ns (no significant difference) p > 0.05.
